# Identification of Attractive Drug Targets in Neglected-Disease Pathogens Using an *In Silico* Approach

**DOI:** 10.1371/journal.pntd.0000804

**Published:** 2010-08-24

**Authors:** Gregory J. Crowther, Dhanasekaran Shanmugam, Santiago J. Carmona, Maria A. Doyle, Christiane Hertz-Fowler, Matthew Berriman, Solomon Nwaka, Stuart A. Ralph, David S. Roos, Wesley C. Van Voorhis, Fernán Agüero

**Affiliations:** 1 Division of Allergy and Infectious Diseases, Department of Medicine, University of Washington, Seattle, Washington, United States of America; 2 Department of Biology and Penn Genomics Institute, University of Pennsylvania, Philadelphia, Pennsylvania, United States of America; 3 Instituto de Investigaciones Biotecnológicas, Universidad Nacional de General San Martín, Buenos Aires, Argentina; 4 Department of Biochemistry and Molecular Biology, Bio21 Molecular Science and Biotechnology Institute, The University of Melbourne, Melbourne, Victoria, Australia; 5 Wellcome Trust Sanger Institute, Hinxton, United Kingdom; 6 Special Programme for Research and Training in Tropical Diseases, World Health Organization, Geneva, Switzerland; McGill University, Canada

## Abstract

**Background:**

The increased sequencing of pathogen genomes and the subsequent availability of genome-scale functional datasets are expected to guide the experimental work necessary for target-based drug discovery. However, a major bottleneck in this has been the difficulty of capturing and integrating relevant information in an easily accessible format for identifying and prioritizing potential targets. The open-access resource TDRtargets.org facilitates drug target prioritization for major tropical disease pathogens such as the mycobacteria *Mycobacterium leprae* and *Mycobacterium tuberculosis*; the kinetoplastid protozoans *Leishmania major*, *Trypanosoma brucei*, and *Trypanosoma cruzi*; the apicomplexan protozoans *Plasmodium falciparum, Plasmodium vivax,* and *Toxoplasma gondii*; and the helminths *Brugia malayi* and *Schistosoma mansoni*.

**Methodology/Principal Findings:**

Here we present strategies to prioritize pathogen proteins based on whether their properties meet criteria considered desirable in a drug target. These criteria are based upon both sequence-derived information (e.g., molecular mass) and functional data on expression, essentiality, phenotypes, metabolic pathways, assayability, and druggability. This approach also highlights the fact that data for many relevant criteria are lacking in less-studied pathogens (e.g., helminths), and we demonstrate how this can be partially overcome by mapping data from homologous genes in well-studied organisms. We also show how individual users can easily upload external datasets and integrate them with existing data in TDRtargets.org to generate highly customized ranked lists of potential targets.

**Conclusions/Significance:**

Using the datasets and the tools available in TDRtargets.org, we have generated illustrative lists of potential drug targets in seven tropical disease pathogens. While these lists are broadly consistent with the research community's current interest in certain specific proteins, and suggest novel target candidates that may merit further study, the lists can easily be modified in a user-specific manner, either by adjusting the weights for chosen criteria or by changing the criteria that are included.

## Author Summary

In cell-based drug development, researchers attempt to create drugs that kill a pathogen without necessarily understanding the details of how the drugs work. In contrast, target-based drug development entails the search for compounds that act on a specific intracellular target—often a protein known or suspected to be required for survival of the pathogen. The latter approach to drug development has been facilitated greatly by the sequencing of many pathogen genomes and the incorporation of genome data into user-friendly databases. The present paper shows how the database TDRtargets.org can identify proteins that might be considered good drug targets for diseases such as African sleeping sickness, Chagas disease, parasitic worm infections, tuberculosis, and malaria. These proteins may score highly in searches of the database because they are dissimilar to human proteins, are structurally similar to other “druggable” proteins, have functions that are easy to measure, and/or fulfill other criteria. Researchers can use the lists of high-scoring proteins as a basis for deciding which potential drug targets to pursue experimentally.

## Introduction

Several strategies exist for the pursuit of drugs to treat neglected tropical diseases. Major approaches can generally be classified as: (A) **label extension**, extending the indications of existing drugs for other conditions to tropical diseases; (B) **piggy-back discovery**, in which the discovery of new drugs is focused on one or a few classes of well-studied and validated targets; and (C) **de novo drug discovery** [1]. These strategies collectively seek to exploit two possible sets of drug targets: those that have been validated in other organisms and diseases, and those that have not – perhaps because they are unique to neglected-disease pathogens – but that nevertheless have potential as novel sites of action.

Since experimental investigations of possible drug targets are time-consuming and expensive, it is worthwhile to conduct *in silico* analyses [2]–[8] to identify the proteins most worthy of experimental follow-up. These analyses consider traits commonly thought to be desirable in a drug target, including essentiality, druggability (whether drug-like molecules are likely to interact with the target), assayability, specificity/selectivity (potential for inhibiting the pathogen without harming the host), and importance in life-cycle stages of the pathogen relevant to human health. Inferring these traits from experimental data is a nontrivial task. For example, guesses at a target's essentiality can be made from gene knockout experiments with the pathogen of interest [9] or related organisms [3], [6], from naturally occurring gene deletions in clinical isolates [10], from microarray and/or proteomic data [11], and/or from metabolic chokepoint (flux balance) studies [12], [13]. Since the best choices are partly a matter of opinion, there is a clear need for databases that are flexible enough to integrate datasets from different sources and to filter these datasets based on the preferences of individual researchers.

To facilitate target-focused analyses for pathogens prioritized by the World Health Organization's Special Programme for Research and Training in Tropical Diseases (TDR), TDRtargets.org [14] was created as a central repository of target-related data. The database may be used for two general scientific tasks: (A) analysis of individual proteins, finding information that relates to their potential as drug targets; and (B) genome-level analysis, sorting and ranking multiple proteins as drug target candidates according to user-specified criteria. The latter task is the main focus of this paper.

TDRtargets.org is designed to facilitate multiple approaches to target prioritization. Users can browse target lists that others have posted (http://tdrtargets.org/published), generate their own lists from standard criteria offered by the database, and/or extend the criteria used to rank prospective targets by uploading files representing additional published or unpublished data. A previous publication [14] has outlined the user interface and concepts underlying the possible queries. In this study, we provide examples of whole-genome prioritization of targets, focusing on key issues for the specific diseases covered. We use these prioritization tools to generate lists of promising drug targets for TDR organisms – lists which provide useful starting points for target characterization in these organisms, as well as illustrate the general utility and versatility of TDRtargets.org in identifying and ranking targets.

## Materials and Methods

### Database Infrastructure

**Figure 1:**
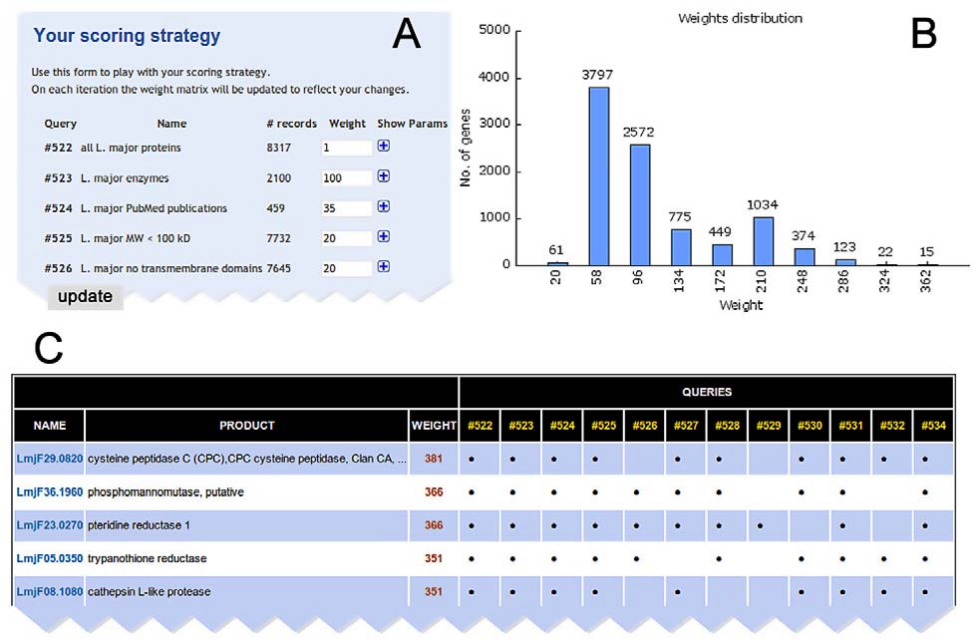
Highlights of the new, improved display of query results in TDRtargets.org. (A) The “Your scoring strategy” panel displays and allows adjustment of weights associated with each criterion. (B) An additional panel shows the distribution of weights among the proteins in the genome. To generate this histogram, all weights in the prioritization strategy were divided into 10 bins; the mean weight for each bin is shown below the x axis. In this example, most proteins had a weight of 0–100, with a small number exceeding 300. (C) Proteins are displayed in descending order of total weight; a grid shows the criteria that were met by each protein.

We have previously described the construction of the TDRtargets.org database, as well as the formulation of searches (queries) to identify proteins meeting criteria of interest and the viewing, saving, and exporting of search results [14]. Since then, while the overall workflow of the database has remained the same, additional genomes and datasets have been included (see below), and several improvements have been implemented on the user interface side of the database. Although users have always been able to perform “weighted union” queries, with different weights (point values) assigned to different user-specified criteria, formulating these queries and viewing and adjusting their results has recently been made more convenient. To construct a weighted union query from the website's target search page, a user (1) selects a pathogen (e.g., *P. falciparum*), (2) selects a criterion (e.g., functional category  =  enzyme) with which to query the pathogen genes, (3) enters a name and a weight for the query in the “Run this query” sub-menu at the bottom of the page, (4) clicks the “Next Query” button, and (5) repeats steps 2 to 4 until the last criterion is entered, at which point the user selects “Run this query” rather than “Next Query.” The search results are displayed on a page where users have the option of changing the previously entered weights for each criterion (Figure 1). (These results are archived on the user's history page, where he/she can combine different subsets of previous queries with the Union function to obtain new ranked target lists.) The presentation of ranked lists has also been revised to display the criteria met by each protein (Figure 1). Further flexibility in data analysis is provided by an option to export the results to a dynamic spreadsheet so that proteins' fulfillment of individual criteria can be viewed and the weights of the criteria can be adjusted offline.

### Using External Data in TDRtargets.org

The TDRtargets.org web application lets users take advantage of datasets obtained externally or in-house. Lists of genes matching user-defined criteria may be saved as text files (each containing a column of gene identifiers – one per line – plus an optional second column for point values, if the targets have been ranked outside of TDRtargets.org) and uploaded at the user's history page. Uploaded lists can be combined with other gene sets from the same organism using any of the history page tools, including ranking by weighted union.

In the present work, a number of target lists meeting different criteria were obtained from external resources, uploaded into TDRtargets.org, and used in various prioritization strategies (see Results), as follows. (A) *T. cruzi* genes with proteomic evidence of expression in amastigotes (at least 2 mass spectra/peptides mapped to the protein) were obtained from TriTrypDB.org [15]. (B) *S. mansoni* genes with evidence for expression at the transcript level (i.e., genes with mapped expressed sequence tags derived from the “egg,” “schistosomula,” and “adult worm” cDNA libraries) were taken from SchistoDB.net [16]. (C) *Drosophila melanogaster* genes associated with abnormal phenotype tags (i.e., “lethal” and “neurophysiological defect”) were taken from FlyBase.org [17]. This list was converted into a list of the corresponding *S. mansoni* orthologs (available from OrthoMCL.org [15]) before uploading into TDRtargets.org.

### Genome Data and Functional Datasets

The current version of the database includes genome data for ten different pathogens (*Brugia malayi, Leishmania major*, *Mycobacterium leprae*, *Mycobacterium tuberculosis*, *Plasmodium falciparum*, *Plasmodium vivax*, *Schistosoma mansoni, Toxoplasma gondii*, *Trypanosoma brucei*, and *Trypanosoma cruzi*) and one endosymbiont bacterium (*Wolbachia,* endosymbiont of *B. malayi*). The depth of data coverage in various functional datasets (searchable at http://tdrtargets.org/search) varies for different organisms; wherever possible, gaps in coverage are compensated for by mapping relevant information from orthologous proteins in other organisms. (For example, protein structure data available for *P. falciparum* proteins were mapped to *P. vivax* proteins.) Ortholog identification on whole genomes was carried out using tools available from OrthoMCL.org [18]. Data recently added to TDRtargets.org include curated data on production of recombinant proteins and activity assays from BRENDA [19]; three-dimensional models of proteins from *B. malayi* and its endosymbiont *Wolbachia*, *M. leprae*, and *S. mansoni,* obtained from ModBase [20]; and phylogenetic information on *Arabidopsis thaliana* (so that users can search for proteins with or without orthologs in plants).

### Ranking Target Genes via Weighted Unions

TDRtargets.org has a flexible ranking system for prioritizing target proteins. In multi-criteria searches, it is possible to take a Boolean intersection of the criteria so that only those proteins with all of the desired traits (e.g., essentiality AND druggability AND assayability, etc.) are selected. However, a protein may lack one or more preferred properties and still be the target of an effective drug (Table 1). Therefore the prioritization queries presented below are devised as weighted unions (see “Database infrastructure” above), in which each criterion is assigned a subjective weight (point value) and targets earn points for each criterion they meet. (Less important and undesirable criteria are given small and negative weights, respectively.) These queries return ranked lists of all potential targets, ordered by cumulative score. Target lists can then be re-ranked, if desired, by changing the weights and/or adding additional criteria (see “Database infrastructure” above).

**Table 1:** Primary targets of drugs used clinically against TDR-prioritized pathogens.

Target	Gene ID	Pathogen	Drug	Molecularweight (kDa)	Trans-membrane domains	PDB structures	ModBase models	Ortholog in humans	Drug-gability	Compound Desirability	Assay-ability
Cytochrome b	*cytb*	*P. falciparum*	Atovaquone	43	8	N	N	Y			N
Cytochrome P-450 14α-demethylase	LmjF11.1100	*L. major*	Fluconazole	54	0	N	Y	Y	0.8	0.43	N
Dihydrofolate reductase	PFD0830w	*P. falciparum*	Pyrimethamine, Cycloguanil/Proguanil	72	0	Y	Y	Y	1	0.56	Y
Dihydrofolate reductase	50.m00016	*T. gondii*	Pyrimethamine	69	0	N	N	Y	0.8	0.56	Y
Dihydropteroate Synthase	ML0224	*M. leprae*	Dapsone	29	ND	Y	Y	N			Y
Dihydropteroate Synthase	PF08_0095	*P. falciparum*	Sulfadoxine	83	0	N	Y	N	0.8		Y
Dihydropteroate Synthase	55.m00011	*T. gondii*	Sulfadiazine	83	0	N	N	N	0.8		N
InhA (NADH-dependent enoyl ACP reductase)	Rv1484	*M. tuberculosis*	Isoniazid	29	0	Y	Y	N	0.7	0.64	Y
Ornithine decarboxylase	Tb11.01.5300	*T. brucei*	Eflornithine (DMFO)	49	0	Y	Y	Y	1	0.43	Y
RNA Polymerase	ML1891	*M. leprae*	Rifampicin	130	ND	N	Y	Y		0.29	Y
RNA Polymerase	Rv00667	*M. tuberculosis*	Rifampicin	129	0	N	N	Y	0.7	0.29	Y

In general, the following might be considered desirable target traits: a low molecular weight and a lack of transmembrane (TM) domains (to favor expression and solubility of recombinant protein), existence of 3D crystal structures and ModBase models (for structure-based drug design), absence of orthologs from humans (to favor selectivity), high druggability and compound desirability scores (0-to-1 scale), and a precedent for assayability. Abbreviations: PDB, Protein Data Bank; Y, yes; N, no; ND, not determined. Note that each target has some desirable features, but few are “perfect.”

### Overview of Queries Presented in This Paper

**Figure 2:**
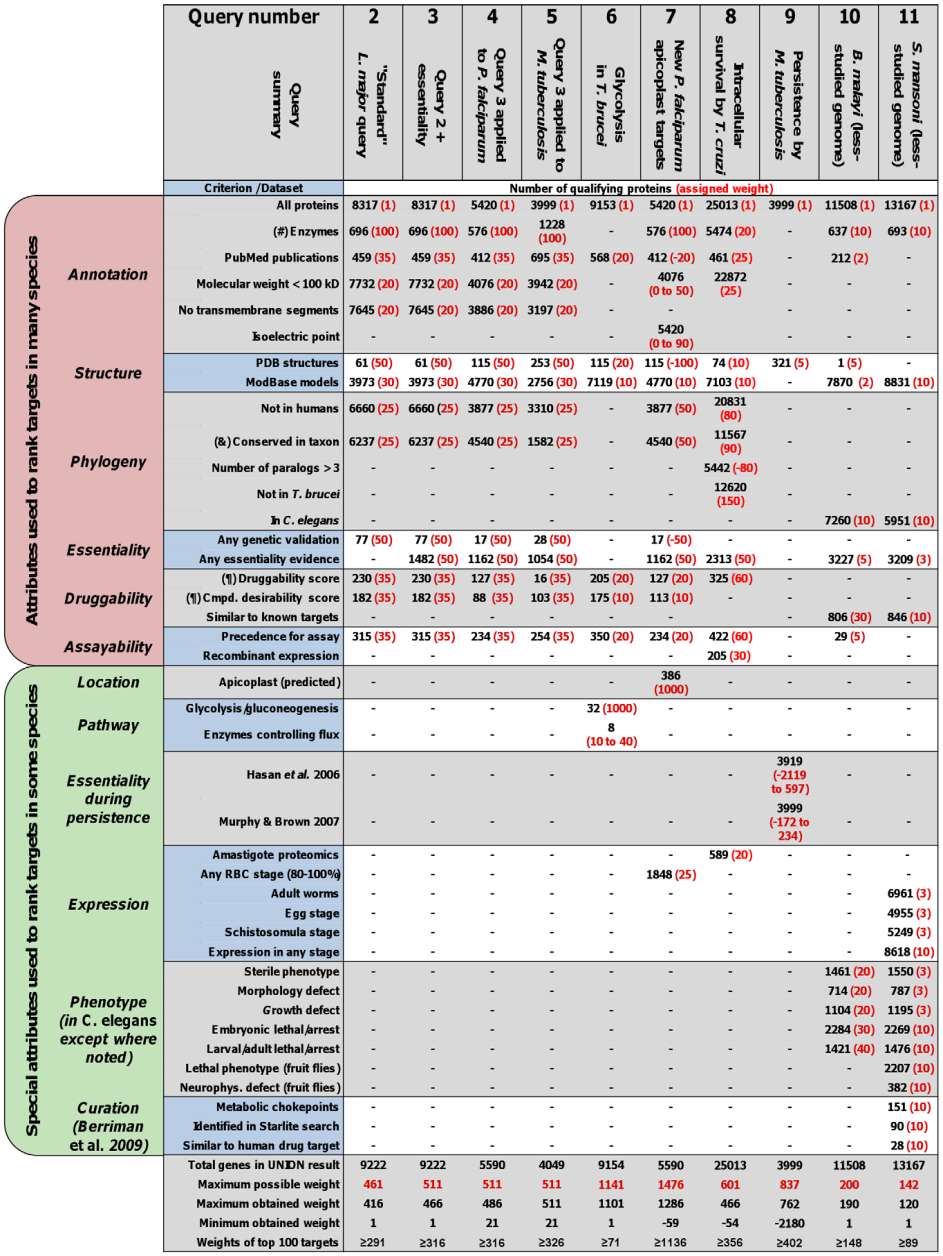
A summary of the multiparameter search queries presented in this study. Ten different queries (Queries 2–11) are listed as individual columns for which the criteria are shown on the left. For each criterion, the number of qualifying proteins from a given pathogen is shown in black and the associated weight is shown in red within parentheses. Symbols: (#) enzymes were selected by combining searches by EC number and by functional category, except for Queries 10 and 11, which were based only on EC number; (&) the conserved-in-taxon criterion refers to the presence of orthologs in *L. major*, *T. brucei*, and *T. cruzi* (Tables 2 and 3), *P. falciparum* and *P. vivax* (Tables 4 and 7), *M. tuberculosis* and *M. leprae* (Table 5), and *L. major* and *T. cruzi* (Table 8); (¶) druggability and compound desirability scores were queried using respective cutoff values of ≥0.6 and >0.3 (Tables 2 to [Table pntd-0000804-t003] [Table pntd-0000804-t004] 5), ≥0.4 and >0.2 (Tables 6 and 7), and ≥0.5 (druggability scores only; Table 8).

The criteria used in generating the lists presented below are summarized in Figure 2. As a starting point, a basic set of criteria of general interest were chosen to frame a “standard” query for identifying targets in *L. major* (see Query 2 in Figure 2). In compiling this basic set of criteria, we included most datasets that are commonly available for organisms with complete genomic information so that the standard query could be easily applied to different pathogens. Queries 3, 4, and 5 of Figure 2 are examples of extending the standard query. Queries 6, 7, 8, and 9 of Figure 2 are framed in a pathogen-specific manner to prioritize target proteins from a particular metabolic pathway, subcellular location, or life-cycle stage. These queries make use of criteria based on external datasets uploaded to TDRtargets.org. (Readers can explore the upload tool at http://tdrtargets.org/history.) Queries 10 and 11 of Figure 2 were based heavily on data obtained by manual curation of the literature [21] and homology/orthology analysis for protein-specific information, illustrating how even incompletely annotated genomes are amenable to target identification. Additional details of these queries are noted below.

## Results

### Searching for Candidate Drug Targets in *Leishmania*

An example of the weighted-union approach to target prioritization (see Methods) is shown in Query 2 of Figure 2, which covers the *Leishmania major* genome. In this example, points are awarded for many of the criteria covered in Table 1, plus some additional conditions. From these criteria a list of prioritized targets is generated (Table 2). Such a list is hardly the final word in *Leishmania* target selection, however. The researchers who generated the list in Table 2 may subsequently decide that, since essentiality data for *Leishmania* genes are very limited, they will consider the presence of an essential ortholog in at least one other organism to be an acceptable predictor of essentiality. Orthologous proteins usually have the same function [22], and several studies indicate that having essential orthologs is predictive of essentiality [23], [24]. The researchers could then amend their initial query so that, for example, 50 additional points are awarded to targets whose orthologs are essential in *C. elegans*, *E. coli*, *M. tuberculosis*, and/or *S. cerevisiae* (the four organisms for which genome-wide essentiality data are available in TDRtargets.org). Such a revision can easily be made by running a new query using the “Any evidence of essentiality in any species” option within the Essentiality subsection of the Search For Genes/Targets page and then using the query history page to find the union of this query and the previous one. The results are similar to but distinct from the previous results (Table 3).

**Table 2:** Preliminary genome-wide prioritization of *Leishmania major* targets.

Ranking	Gene_name	Gene product	Weight
1	LmjF29.0820	cysteine peptidase C (CPC),CPC cysteine peptidase, Clan CA, family C1, Cathepsin B-like	416
2	LmjF05.0350	trypanothione reductase	386
2	LmjF06.0860	dihydrofolate reductase-thymidylate synthase	386
2	LmjF23.0050	cyclophilin, putative,peptidyl-prolyl cis-trans isomerase, putative	386
2	LmjF25.0910	cyclophilin a	386
2	LmjF06.0120	cyclophilin	386
2	LmjF18.0270	protein kinase, putative,glycogen synthase kinase, putative	386
8	LmjF36.1960	phosphomannomutase, putative	366
8	LmjF23.0270	pteridine reductase 1	366
10	LmjF30.2970	glyceraldehyde 3-phosphate dehydrogenase, glycosomal	351
10	LmjF12.0220	hydroxyacylglutathione hydrolase, putative,glyoxalase II, putative	351
10	LmjF24.0850	triosephosphate isomerase	351
13	LmjF27.1870	trypanothione synthetase, putative	341
13	LmjF06.0560	deoxyuridine triphosphatase, putative,dUTP diphosphatase	341
15	LmjF21.0250	hexokinase, putative	336
15	LmjF25.1320	serine/threonine protein phosphatase, putative	336
15	LmjF19.0550	methionine aminopeptidase, putative,metallo-peptidase, Clan MG, Family M24	336
15	LmjF34.1260	mitochondrial DNA polymerase I protein A, putative	336
15	LmjF30.0880	adenosine kinase, putative	336
15	LmjF33.1630	cyclophilin, putative	336
15	LmjF10.0890	FKBP-type peptidyl-prolyl cis-trans isomerase, putative	336
15	LmjF04.1160	fructose-1,6-bisphosphatase, cytosolic, putative	336
15	LmjF23.0950	cytosolic leucyl aminopeptidase,metallo-peptidase, Clan MF, Family M17	336
15	LmjF32.1580	phosphomannose isomerase, putative	336
25	LmjF36.2380	sterol 24-c-methyltransferase, putative	326
25	LmjF36.2390	sterol 24-c-methyltransferase, putative	326

Top targets according to the criteria shown in Query 2 of Figure 2. Complete genome-wide rankings for this example and all other examples discussed in the paper (Tables 3–11) are available online at http://www.tdrtargets.org/published/browse/366. Please note that multiple targets often receive the same total weight, and that the order in which these “tied” targets are displayed has no significance.

**Table 3 pntd-0000804-t003:** Revised *L. major* rankings after incorporating an essential-in-other-species criterion.

Ranking	Gene name	Gene product	Weight
1	LmjF29.0820	cysteine peptidase C (CPC),CPC cysteine peptidase, Clan CA, family C1, Cathepsin B-like	466
2	LmjF05.0350	trypanothione reductase	436
2	LmjF06.0860	dihydrofolate reductase-thymidylate synthase	436
2	LmjF23.0050	cyclophilin, putative,peptidyl-prolyl cis-trans isomerase, putative	436
2	LmjF25.0910	cyclophilin a	436
2	LmjF06.0120	cyclophilin	436
2	LmjF18.0270	protein kinase, putative,glycogen synthase kinase, putative	436
8	LmjF36.1960	phosphomannomutase, putative	416
9	LmjF30.2970	glyceraldehyde 3-phosphate dehydrogenase, glycosomal	401
9	LmjF24.0850	triosephosphate isomerase	401
11	LmjF21.0250	hexokinase, putative	386
11	LmjF25.1320	serine/threonine protein phosphatase, putative	386
11	LmjF19.0550	methionine aminopeptidase, putative,metallo-peptidase, Clan MG, Family M24	386
11	LmjF34.1260	mitochondrial DNA polymerase I protein A, putative	386
11	LmjF30.0880	adenosine kinase, putative	386
11	LmjF33.1630	cyclophilin, putative	386
11	LmjF32.1580	phosphomannose isomerase, putative	386
18	LmjF35.0030	pyruvate kinase, putative	366
18	LmjF36.1260	fructose-1,6-bisphosphate aldolase	366
*18*	*LmjF35.0020*	*pyruvate kinase, putative*	*366*
*18*	*LmjF16.1540*	*DNA polymerase I alpha catalytic subunit, putative*	*366*
*18*	*LmjF20.0100*	*phosphoglycerate kinase C, glycosomal*	*366*
*18*	*LmjF18.0990*	*UTP-glucose-1-phosphate uridylyltransferase 2, putative*	*366*
*18*	*LmjF18.0090*	*alpha glucosidase II subunit, putative*	*366*
*18*	*LmjF21.1080*	*cell division protein kinase 2,cdc2-related kinase*	*366*
*18*	*LmjF26.0140*	*adenine phosphoribosyltransferase*	*366*
*18*	*LmjF12.0530*	*glucose-6-phosphate isomerase*	*366*
18	LmjF23.0270	pteridine reductase 1	366
*18*	*LmjF33.1690*	*DNA polymerase delta catalytic subunit, putative*	*366*
*18*	*LmjF28.2280*	*DNA topoisomerase ii*	*366*

Top targets according to the criteria shown in Query 3 of Figure 2. Italicized targets are those that were not top-ranked in the list shown in Table 2.

Now consider a more drastic revision of the *Leishmania* search: application of the previous criteria (Figure 2, Query 3) to two other pathogens, namely *P. falciparum* and *M. tuberculosis*. This too is readily done within TDRtargets.org – there is a “Change species” option on the Query History page – again highlighting the ease of modifying previous searches. While use of exactly the same criteria to prioritize targets in different species might seem naïve, the results (Tables 4 and 5) are instructive. First of all, the top-ranked proteins of each species are rather different, showing that this search strategy is sensitive to species differences, as opposed to being unalterably biased toward the same proteins in every species. Second, many of the top-ranked targets in each species appear to be appealing options. For example, the three top-scoring targets from each species – dihydrofolate reductase/thymidylate synthase, enoyl-ACP reductase, and triose-phosphate isomerase in *P. falciparum* and enoyl-ACP reductase (InhA), glutamine synthetase, and 5-enolpyruvylshikimate-3-phosphate synthase (AroA) in *M. tuberculosis* – have all attracted interest as proven or prospective targets [25]–[30]. It is interesting that legitimate candidates such as these rise to the top of the target rankings despite certain quirks of this “one set of criteria fits all species” example. In the *M. tuberculosis* prioritization, for instance, many of the top-ranked targets are essential even though the genome-wide mutagenesis data available for this species were not queried. Thus, although these lists are imperfect, they generally suggest that rational choices of criteria lead to plausible and informative rankings of target desirability across species.

**Table 4 pntd-0000804-t004:** Application of standard search criteria to *P. falciparum*.

Ranking	Gene name	Gene product	Weight
*1*	*PFD0830w*	*bifunctional dihydrofolate reductase-thymidylate synthase*	*486*
*2*	*PFF0730c*	*enoyl-acyl carrier reductase*	*461*
*3*	*PF14_0378*	*triose-phosphate isomerase*	*451*
4	PF11_0282	deoxyuridine 5′-triphosphate nucleotidohydrolase, putative	436
4	PFC0975c	PFCYP19, cyclophilin, peptidyl-prolyl cis-trans isomerase	436
4	PF10_0289	adenosine deaminase, putative	436
4	PFI1105w	Phosphoglycerate kinase	436
4	PF14_0192	glutathione reductase	436
4	PFE1050w	adenosylhomocysteinase(S-adenosyl-L-homocysteine hydrolase)	436
10	PFD0980w	holo-(acyl-carrier protein) synthase, putative	426
10	PFF1105c	chorismate synthase	426
12	PFF0160c	dihydroorotate dehydrogenase, mitochondrial precursor	416
12	PF14_0053	ribonucleotide reductase small subunit	416
12	PF14_0425	fructose-bisphosphate aldolase	416
15	PF14_0641	1-deoxy-D-xylulose 5-phosphate reductoisomerase	411
15	PFB0505c	beta-ketoacyl-acyl carrier protein synthase III precursor, putative	411
17	PF14_0164	NADP-specific glutamate dehydrogenase	401
17	PF14_0142	serine/threonine protein phosphatase, putative	401
17	PF11_0377	casein kinase 1	401
17	PFL2275c	70 kDa peptidylprolyl isomerase, putative	401
17	PF13_0287	adenylosuccinate synthetase	401
17	PF10_0121	hypoxanthine phosphoribosyltransferase	401
23	PF08_0095	dihydropteroate synthetase	391
24	PFE1360c	methionine aminopeptidase, putative	386
24	PF14_0327	methionine aminopeptidase, type II, putative	386
24	PFF1155w	hexokinase	386
24	PFI1110w	glutamate—ammonia ligase (glutamine synthetase), putative	386
24	PFC0525c	glycogen synthase kinase, putative	386
24	PF10_0150	methionine aminopeptidase, putative	386
24	PFI1170c	thioredoxin reductase	386
24	PF11_0164	peptidyl-prolyl cis-trans isomerase	386

Top targets for *P. falciparum* according to the search criteria shown in Query 4 of Figure 2.

Targets mentioned in the text are italicized.

**Table 5 pntd-0000804-t005:** Application of standard search criteria to *M. tuberculosis*.

Ranking	Gene name	Gene product	Weight
*1*	*Rv1484*	*nadh-dependent enoyl-[acyl-carrier-protein] reductase inha (nadh-dependent enoyl-acp reductase)*	*511*
*2*	*Rv2220*	*glutamine synthetase glna1 (glutamine synthase) (gs-i)*	*451*
*3*	*Rv3227*	*3-phosphoshikimate 1-carboxyvinyltransferase aroa (5-enolpyruvylshikimate-3-phosphate synthase) (epsp synthase) (epsps)*	*426*
4	Rv3581c	probable 2c-methyl-d-erythritol 2,4-cyclodiphosphate synthase ispf (mecps)	406
4	Rv2763c	dihydrofolate reductase dfra (dhfr) (tetrahydrofolate dehydrogenase)	406
4	Rv2537c	3-dehydroquinate dehydratase arod (aroq) (3-dehydroquinase) (type ii dhqase)	406
4	Rv3602c	probable pantoate—beta-alanine ligase panc (pantothenate synthetase) (pantoate activating enzyme)	406
4	Rv1293	probable diaminopimelate decarboxylase lysa (dap decarboxylase)	406
9	Rv0533c	3-oxoacyl-[acyl-carrier-protein] synthase iii fabh (beta-ketoacyl-acp synthase iii) (kas iii)	401
9	Rv2861c	probable methionine aminopeptidase mapb (map) (peptidase m)	401
9	Rv2860c	probable glutamine synthetase glna4 (glutamine synthase) (gs-ii)	401
9	Rv2222c	probable glutamine synthetase glna2 (glutamine synthase) (gs-ii)	401
9	Rv1878	probable glutamine synthetase glna3 (glutamine synthase) (gs-i)	401
14	Rv2870c	probable 1-deoxy-d-xylulose 5-phosphate reductoisomerase dxr (dxp reductoisomerase) (1-deoxyxylulose-5-phosphate reductoisomeras	396
15	Rv3566c	arylamine n-acetyltransferase nat (arylamine acetylase)	391
15	Rv1207	probable dihydropteroate synthase 2 folp2 (dhps 2) (dihydropteroate pyrophosphorylase 2)	391
15	Rv2225	probable 3-methyl-2-oxobutanoate hydroxymethyltransferase panb	391
15	Rv3628	inorganic pyrophosphatase ppa (pyrophosphate phospho-hydrolase) (ppase) (inorganic diphosphatase) (diphosphate phospho-hydrolase	391
15	Rv3014c	probable dna ligase [nad dependent] liga (polydeoxyribonucleotide synthase [nad+])	391
20	Rv1483	3-oxoacyl-[acyl-carrier protein] reductase fabg1 (3-ketoacyl-acyl carrier protein reductase) (mycolic acid biosynthesis a protei	386
20	Rv1007c	probable methionyl-trna synthetase mets (metrs) (methionine—trna ligase)	386
20	Rv0014c	transmembrane serine/threonine-protein kinase b pknb (protein kinase b) (stpk b)	386
23	Rv2428	alkyl hydroperoxide reductase c protein ahpc (alkyl hydroperoxidase c)	381
24	Rv0764c	cytochrome p450 51 cyp51 (cypl1) (p450-l1a1) (sterol 14-alpha demethylase) (lanosterol 14-alpha demethylase) (p450-14dm)	376

Top targets for *M. tuberculosis* according to the search criteria shown in Query 5 of Figure 2. Targets mentioned in the text are italicized.

### *T. brucei* and *P. falciparum*: Metabolic Pathway- and Organelle-Specific Targets

While TDRtargets.org integrates numerous datasets relevant to target prioritization, it cannot possibly anticipate every possible prioritization strategy that could be used by any given researcher. Accordingly, users can upload (weighted or unweighted) lists of targets meeting any criteria for which they have relevant data; these may then be combined with other queries covered by TDRtargets.org. Supplementation of standard TDRtargets.org criteria with a user-provided criterion is illustrated in the following example. Researchers specializing in the *T. brucei* glycolytic pathway are convinced that this pathway is essential in these parasites and wish to rank the enzymes within this pathway for their suitability as drug targets. Since they already assume the pathway to be essential and know glycolysis is also present in host cells, they may not address these issues in their search criteria, but may instead award points as listed in Query 6 of Figure 2. The query shown there combines criteria addressing integral TDRtargets.org data (such as availability of structural models) with a user-generated list of “bonus points” to some *T. brucei* enzymes in proportion to their relative control over the glycolytic flux [31]. The rationale for such a scoring might be that the greater an enzyme's flux control, the less completely it must be inhibited for flux through the entire pathway to be affected (and thus the better a target it is). In this example, the inclusion of flux control as a criterion lifts the two glycosomal orthologs of glyceraldehyde-3-phosphate dehydrogenase, the enzyme with the highest control coefficient, to the top of the priority list (Table 6). The recent genetic validation of this enzyme [32] likewise identifies it as a possible target of interest. Interestingly, hexokinase was thought to have a much lower control coefficient [31] but may also have promise as a drug target [33].

**Table 6 pntd-0000804-t006:** Prioritization of glycolytic enzymes in *T. brucei*.

Ranking	Gene name	Gene product	Weight
1	Tb927.1.700	phosphoglycerate kinase	1101
1	Tb11.02.3210	triosephosphate isomerase	1101
*1*	*Tb927.6.4300*	*glyceraldehyde 3-phosphate dehydrogenase, glycosomal*	*1101*
*1*	*Tb927.6.4280*	*glyceraldehyde 3-phosphate dehydrogenase, glycosomal*	*1101*
5	Tb927.1.710	phosphoglycerate kinase	1081
5	Tb09.211.0540	fructose-1,6-bisphosphate, cytosolic	1081
*5*	*Tb10.70.5800*	*hexokinase*	*1081*
*5*	*Tb10.70.5820*	*hexokinase*	*1081*
9	Tb927.3.3270	ATP-dependent phosphofructokinase,6-phospho-1-fructokinase	1071
9	Tb10.70.1370	fructose-bisphosphate aldolase, glycosomal	1071
9	Tb927.1.3830	glucose-6-phosphate isomerase, glycosomal	1071
9	Tb10.70.4740	enolase	1071
13	Tb927.1.720	phosphoglycerate kinase	1061
13	Tb10.6k15.3850	glyceraldehyde 3-phosphate dehydrogenase, cytosolic	1061
15	Tb927.3.4390	dihydrolipoamide dehydrogenase, putative	1051
15	Tb11.01.8100	enolase, putative	1051
15	Tb10.61.2680	pyruvate kinase 1	1051
15	Tb09.211.1370	glyceraldehyde-3-phosphate dehydrogenase, putative	1051
15	Tb927.8.7380	dihydrolipoamide dehydrogenase, point mutation,acetoin dehydrogenase e3 component, putative	1051
15	Tb927.4.5040	dihydrolipoamide dehydrogenase, putative	1051
15	Tb927.5.3580	phosphoglycerate mutase protein, putative	1051
15	Tb11.01.8470	dihydrolipoyl dehydrogenase	1051
23	Tb10.6k15.2620	2,3-bisphosphoglycerate-independent phosphoglycerate mutase	1031
23	Tb927.8.2520	acetyl-CoA synthetase, putative	1031
25	Tb927.6.3050	aldehyde dehydrogenase family, putative	1011
25	Tb10.6k15.3080	dihydrolipoamide acetyltransferase precursor, putative	1011
25	Tb10.70.5380	dihydrolipoamide acetyltransferase, putative	1011
25	Tb10.389.0890	pyruvate dehydrogenase E1 component alpha subunit, putative	1011
25	Tb927.3.2030	acylphosphatase, putative	1011
25	Tb927.6.4210	aldehyde dehydrogenase, putative	1011
25	Tb927.3.1790	pyruvate dehydrogenase E1 beta subunit, putative	1011
32	Tb10.70.2900	2-oxoisovalerate dehydrogenase beta subunit, mitochondrial precursor, putative	1001

Top targets according to the search criteria shown in Query 6 of Figure 2. Targets mentioned in the text are italicized.

The next scenario also employs a user-provided list, which in this case permits scrutiny of a specific organelle rather than a specific metabolic pathway. Consider a newly independent crystallographer with a special interest in *Plasmodium* apicoplasts, which are absent from the human host and thus are likely to contain many appealing drug targets [34]. The PlasmoAP algorithm [35] predicts that 485 proteins are localized to the apicoplast; the user can download this list from PlasmoDB.org [36], manually delete proteins that seem unlikely to reside in the apicoplast, and then upload the modified list to TDRtargets.org. In sorting through the ∼400 proteins likely to reside in the apicoplast, the user may decide to minimize competition with labs already working on apicoplast biology by penalizing well-studied proteins (e.g., subtracting 100 points from targets whose 3D structures have already been solved) while rewarding other desirable characteristics such as those discussed above (likely essentiality, lack of orthologs in humans, etc.). Finally, a previous publication [37] has convinced the hypothetical user that a low molecular weight and low isoelectric point (pI) improve the odds of successful expression of soluble *Plasmodium* proteins, so those factors are weighted accordingly (Query 7 of Figure 2). The most highly ranked proteins in this example (Table 7) include some proteins (e.g., pseudouridine synthetase and cysteine desulfurase) that are rarely mentioned in the *Plasmodium* literature, consistent with this researcher's desire to explore truly novel target options.

**Table 7 pntd-0000804-t007:** Possible novel drug targets in *P. falciparum* apicoplasts.

Ranking	Gene name	Gene product	Weight
1	PF13_0176	apurinic/apyrimidinic endonuclease Apn1	1286
2	PFA0225w	LytB protein	1276
3	MAL13P1.221	aspartate carbamoyltransferase	1261
*3*	*PFB0890c*	*pseudouridine synthetase, putative*	*1261*
*3*	*PF07_0068*	*cysteine desulfurase, putative*	*1261*
3	PF10_0221	GcpE protein	1261
7	PF14_0063	ATP-dependent Clp protease, putative	1256
7	PF11_0270	threonine — tRNA ligase, putative	1256
9	PFI1240c	prolyl-t-RNA synthase, putative	1241
9	PFL1120c	DNA GyrAse a-subunit, putative	1241
9	PF10_0053	tRNA ligase, putative	1241
12	PFL0770w	seryl-tRNA synthetase, putative	1236
12	PF07_0129	ATP-dept. acyl-coa synthetase	1236
12	PFE0475w	asparagine — t RNA ligase, putative	1236
12	PF10_0363	pyruvate kinase, putative	1236
12	PF13_0354	alanine—tRNA ligase, putative	1236
12	PFB0695c	acyl-CoA synthetase	1236
12	PFE0205w	ATP-dependent helicase, putative	1236
12	PF13_0077	DEAD box helicase, putative	1236
20	MAL13P1.281	glutamate—tRNA ligase, putative	1231
20	PF14_0348	ATP-dependent Clp protease proteolytic subunit, putative	1231
22	PF14_0112	POM1, putative	1221
23	PF11_0174	hypothetical protein	1216
23	PF08_0108	pepsinogen, putative	1216
23	PFL2395c	dimethyladenosine transferase, putative	1216
23	PFE0195w	P-type ATPase, putative	1216

Top targets according to the search criteria shown in Query 7 of Figure 2. Proteins shown are likely to (A) be good drug targets, (B) be amenable to crystallization, and (C) reside in the apicoplast. Targets mentioned in the text are italicized.

### *Trypanosoma cruzi*: Candidate Targets Associated with an Intracellular Lifestyle

Unlike the bloodstream trypomastigotes of African Trypanosomes (Salivaria), the *T. cruzi* (Stercoraria) bloodstream forms do not replicate, and instead invade cells. In this parasitic strategy, which is shared with *Leishmania* spp., the replicative amastigotes are the intracellular parasite forms that persist and maintain the infection. Given the early evolutionary divergence of Salivarian trypanosomes [38] and the different strategies used by Salivarian and Stercorarian parasites to mount and maintain an infection, these groups of parasites may exhibit numerous instances of (A) gene loss and (B) gene duplications followed by neofunctionalization [39]. Proteins that are orthologous between *T. cruzi* and *Leishmania* but that lack *T. brucei* counterparts may represent proteins vital to intracellular survival and/or growth, which could be excellent targets for drug development.

To look for such proteins, we used a general strategy similar to that used for *Leishmania* (see Query 3 of Figure 2) but now focused on *T. cruzi*, with an extra phylogeny-based restriction: additional weight was added to proteins that have been conserved in *Leishmania* and *T. cruzi* but that have been lost or substantially changed in *T. brucei.* The attributes and weights used in this query are shown in Query 8 of Figure 2. The strategy also relies on proteomic evidence of expression in intracellular amastigotes [40]. However, because the proteomic data have a low coverage of the proteome, only a moderate weight has been assigned to this criterion. (This illustrates users' ability to assign relative weights based not only on which characteristics they consider predictive of target desirability, but also on their confidence that available experimental datasets accurately reflect those characteristics.)

The results of this prioritization of *T. cruzi* targets are shown in Table 8. Because of the hybrid nature of the strain used to sequence the genome of *T. cruzi*, the list is somewhat redundant: most single copy genes appear twice in all genome databases. The top 32 targets include representatives of validated pathways – ergosterol biosynthesis, as represented by sterol C-24 reductase, and glycolysis, as represented by glucokinase – and other interesting alternatives for drug development. As suggested above, glycolysis is an essential pathway in trypanosomes, and the glycosome-localized glucokinase has attracted interest as a possible target since it was discovered in the sequenced *Leishmania* and *T. cruzi* genomes [41]. On the other hand, the top- ranked sterol C-24 reductase provides a good example of the attractiveness of the phylogenetic criteria used in this strategy. The ergosterol biosynthesis pathway is also present in *T. brucei*, although it is not essential for the bloodstream forms, which scavenge sterols from the host [42]. This highly ranked C-24 reductase belongs to the OrthoMCL ortholog group OG4_16908 (OrthoMCL version 4), which contains orthologs from the genomes of *T. cruzi, L. major*, and yeast (ERG4). However, this enzyme is apparently absent in the genomes of *T. brucei TREU927*, *T. brucei gambiense*, *T. vivax*, *and T. congolense.* In yeast, ERG4 catalyzes the final step in ergosterol biosynthesis, and although mutants are viable, they show a number of abnormal phenotypes and decreased fitness (see http://www.yeastgenome.org/cgi-bin/locus.fpl?locus=ERG4).

**Table 8 pntd-0000804-t008:** Possible *T. cruzi* drug targets likely to be important in intracellular survival.

Ranking	Gene name	Gene product	Weight
1	Tc00.1047053508111.30	glutamate dehydrogenase, putative	466
*1*	*Tc00.1047053510879.80*	*serine acetyltransferase, putative*	*466*
*1*	*Tc00.1047053504013.40*	*serine acetyltransferase, putative*	*466*
4	Tc00.1047053507875.20	glutamate dehydrogenase, putative	456
*5*	*Tc00.1047053511277.600*	*hypothetical protein, conserved*	*436*
*6*	*Tc00.1047053510187.100*	*glucokinase 1, putative*	*421*
7	Tc00.1047053503745.30	ascorbate-dependent peroxidase, putative	416
8	Tc00.1047053506193.60	ascorbate-dependent peroxidase, putative	406
8	Tc00.1047053507993.160	hypothetical protein, conserved	406
8	Tc00.1047053503749.5	pyrroline-5-carboxylate synthetase-like protein, putative	406
8	Tc00.1047053508699.120	dipeptidyl-peptidase, putative	406
8	Tc00.1047053509205.120	hypothetical protein, conserved	406
*13*	*Tc00.1047053509073.70*	*phosphatidate cytidylyltransferase-like protein, putative*	*396*
13	Tc00.1047053508601.141	dipeptidyl-peptidase, putative	396
13	Tc00.1047053508707.140	phosphatidate cytidylyltransferase-like protein, putative	396
*16*	*Tc00.1047053509287.20*	*protein kinase, putative*	*386*
16	Tc00.1047053506577.60	hypothetical protein, conserved	386
16	Tc00.1047053506953.30	protein kinase, putative	386
*19*	*Tc00.1047053506839.60*	*tyrosine specific protein phosphatase, putative*	*381*
19	Tc00.1047053506737.20	protein kinase, putative	381
19	Tc00.1047053511277.210	peroxisomal enoyl-coa hydratase, putative	381
19	Tc00.1047053508717.10	tyrosine specific protein phosphatase, putative	381
19	Tc00.1047053508637.90	phosphoglucomutase, putative	381
24	Tc00.1047053506725.20	hypothetical protein, conserved	376
24	Tc00.1047053508461.80	prostaglandin F2alpha synthase	376
*24*	*Tc00.1047053506577.120*	*sterol C-24 reductase, putative*	*376*
24	Tc00.1047053511761.60	hypothetical protein, conserved	376
24	Tc00.1047053507617.9	prostaglandin F2alpha synthase	376
24	Tc00.1047053508955.20	hypothetical protein, conserved	376
24	Tc00.1047053507089.170	hypothetical protein, conserved	376
24	Tc00.1047053506679.130	hypothetical protein, conserved	376
24	Tc00.1047053507709.60	hypothetical protein, conserved	376

Top targets according to the search criteria shown in Query 8 of Figure 2. Targets mentioned in the text are italicized.

Another top-ranking target in Table 8 is the *T. cruzi* serine acetyltransferase (TcSAT), involved in the *de novo* synthesis of cysteine, which is present in *Leishmania* and *T. cruzi* and absent in *T. brucei* [43]. Cysteine in these parasites is important for the biosynthesis of polyamines and for antioxidant metabolism based on trypanothione, the trypanosome equivalent of glutathione. Inhibitors of the *E. coli* SAT enzyme have recently been shown to inhibit the growth of *Entamoeba histolytica,* another pathogen that is highly sensitive to oxidative stress [44].

Other interesting targets in this list include a putative amine oxidase (Tc00.1047053511277.600) which further analysis shows is conserved in several *Leishmania* species but absent in sequenced *T. brucei* subspecies; a putative phosphatidate cytidylyltransferase (Tc00.1047053509073.70) that belongs to an ortholog group with a very restricted phylogenetic distribution (OG4_29276), with members from many *Leishmania* species with complete genomes, *Entamoeba histolytica* (another pathogen), and two non-pathogenic species (*Thalassiosira pseudonana* and *Aquifex aeolicus*); a protein kinase (Tc00.1047053509287.20) whose yeast orthologs regulate endocytosis through the organization and function of the actin cytoskeleton; and a tyrosine protein phosphatase (Tc00.1047053506839.60) that also shows an unusual phylogenetic distribution, being almost exclusively present in *T. cruzi, Leishmania* spp., and metazoa.

### *Mycobacterium tuberculosis*: Exploiting Previous Prioritizations

Previous target prioritization efforts [2]–[8] raise the question of how these efforts should be viewed in relation to TDRtargets.org. We consider TDRtargets.org to be complementary to others' prioritization work, and anticipate that it can be used to combine and apply the ranking methods of other target identification efforts. For instance, a recent paper on *M. tuberculosis* by Hasan and colleagues [4] provided an excellent synthesis of experimental data to rank targets by persistence in dormant stages. These data (available in [4] as Supplemental Dataset S1, and also at http://tdrtargets.org/published/browse/379) can be easily interrogated and combined with other queries using TDRtargets.org. For example, while Hasan *et al.*'s rankings considered proteins essential for growth on defined medium *in vitro* [45], [46], they did not reward proteins thought to be essential for growth in macrophages or in the infection of mice [47], [48], which may well be very relevant to human infection. In addition, because Hasan *et al.* awarded points to proteins with solved crystal structures, it seems apt to give points to proteins whose structures have been solved during the four years that have elapsed since the original analysis was published. TDRtargets.org was therefore used to make a few modifications to one set of Hasan *et al.* 's rankings: the set that emphasized targets' likely importance in persistent-stage disease. We uploaded a modified version of this list that excluded points for PDB structures, then gave additional points to all genes represented in the Protein Data Bank of crystal structures [49] as of April 2010. To these subtotals, we added points based on an analysis of latent-stage infections by Murphy & Brown [7]. In that analysis, genes were given upregulation and downregulation scores based on their expression in various models of dormancy, thus offering a distinct estimate of genes' importance during latency, and “attenuation” scores based on the effect of gene knockouts on growth in various contexts, including the macrophage and mouse studies noted above. (See “Additional file 1” from [7]; see also http://tdrtargets.org/published/browse/383.) The combined input of the two previous studies was thus used to create a “consensus list” (Table 9) that might be considered superior to either one alone. Combining the two previous analyses could also be done off-line using spreadsheets, but performing these operations within TDRtargets.org is considerably faster and facilitates retrieval of TDRtargets.org-compiled information on each individual target. Naturally, our “consensus list” reflects the limitations of the previous analyses, e.g., the low rankings of important persistent-stage proteins such as Rv0470c (mycolic acid synthase, PcaA) and Rv2583c (GTP pyrophosphokinase, RelA), as discussed by Hasan et al. [4].

**Table 9 pntd-0000804-t009:** Leading persistent-stage *M. tuberculosis* targets.

Ranking	Gene name	Gene product	Weight
1	Rv0885	conserved hypothetical protein	762
2	Rv3290c	probable l-lysine-epsilon aminotransferase lat (l-lysine aminotransferase) (lysine 6-aminotransferase)	752
3	Rv2004c	conserved hypothetical protein	717
4	Rv2780	secreted l-alanine dehydrogenase ald (40 kda antigen) (tb43)	714
5	Rv2628	hypothetical protein	679
6	Rv2626c	conserved hypothetical protein	657
6	Rv2623	conserved hypothetical protein tb31.7	657
8	Rv3340	probable o-acetylhomoserine sulfhydrylase metc (homocysteine synthase) (o-acetylhomoserine (thiol)-lyase) (oah sulfhydrylase) (o	631
9	Rv2497c	probable pyruvate dehydrogenase e1 component (alpha subunit) pdha (pyruvate decarboxylase) (pyruvate dehydrogenase) (pyruvic deh	615
10	Rv2629	conserved hypothetical protein	613
11	Rv2627c	conserved hypothetical protein	610
12	Rv3130c	conserved hypothetical protein	605
13	Rv2035	conserved hypothetical protein	602
14	Rv2624c	conserved hypothetical protein	601
15	Rv0678	conserved hypothetical protein	599
16	Rv2032	conserved hypothetical protein acg	596
17	Rv1813c	conserved hypothetical protein	594
18	Rv3131	conserved hypothetical protein	591
19	Rv2630	hypothetical protein	580
20	Rv0251c	heat shock protein hsp (heat-stress-induced ribosome-binding protein a)	579
21	Rv1285	probable sulfate adenylyltransferase subunit 2 cysd	576
22	Rv2830c	conserved hypothetical protein	569
23	Rv0275c	possible transcriptional regulatory protein (possibly tetr-family)	566
24	Rv2711	iron-dependent repressor and activator ider	565
25	Rv3126c	hypothetical protein	556

Top targets according to the search criteria shown in Query 9 of Figure 2. In essence, previous analyses by Hasan et al. [4] and Murphy & Brown [7] were combined.

### Helminths: The Importance of Homology

Since many valuable helminth datasets are only starting to emerge, our attempts to prioritize helminth targets required some analysis beyond the standard TDRtargets.org queries. For example, *B. malayi* and *S. mansoni* proteins are not yet scored for druggability in TDRtargets.org, so we assessed their druggability by comparing their amino acid sequences to those of known drug targets in the StARLITe/ChEMBL database [50]. The sequence similarity analysis was performed using BLAST; a helminth protein was considered druggable if (A) it is ≥80% of the length of the corresponding druggable target, (B) it has an amino-acid sequence that aligns with ≥80% of the druggable target, and (C) the BLAST expectation value of the alignment is less than 10^−10^ (database size: 11,508 genes for *B. malayi*, 13,331 genes for *S. mansoni*). In addition, proteins' functional importance in helminths was inferred from knockout data taken from their orthologs in *C. elegans* and *D. melanogaster* (see Materials and Methods and Queries 10 and 11 of Figure 2). Being able to connect the helminth proteins to similar proteins in other species was thus critical in allowing us to evaluate their potential as drug targets.

Our strategy of relying heavily on orthology and sequence similarity to rank helminth targets is broadly similar to those used by Kumar *et al.* [6] to rank *Brugia* targets and by Caffrey *et al.* [3] to rank *Schistosoma* targets. However, these authors sought targets that met each of several desired criteria (Boolean “AND”); for example, Kumar *et al.* only considered *Brugia* proteins with orthologs in *C. elegans* but not in humans, and whose absence causes deleterious phenotypes (according to RNAi of *C. elegans* orthologs). In contrast, we again used the “weighted union” approach to avoid premature elimination of any proteins from consideration as targets. Kumar *et al.* also took a distinct approach to druggability, rewarding proteins with domains targeted by compounds obeying the Lipinski “Rule of 5” [51] and having EC numbers associated with druggability. Additionally, Kumar *et al.* penalized proteins for hydropathicity (which reduces the ease of recombinant expression) and rewarded them for being expressed (according to a small dataset of expressed sequence tags, or ESTs, encompassing 250 genes); in contrast, we gave additional points to all proteins having EC numbers (and therefore presumed to be enzymes), 3D structural models, and/or bibliographic references.

A comparison of our helminth prioritizations (Tables 10 and 11) with those of Kumar *et al.* [6] and Caffrey *et al.* [3] reveals relatively little concordance. Among our top 200 *Brugia* targets, none are also among the top 200 as ranked by Kumar *et al*. (see Supplementary Table S1 of [6], also available at http://tdrtargets.org/published/browse/282). This lack of overlap is likely due in part to (A) our emphasis on druggability, as inferred from sequence similarity against targets in the ChEMBL database, and (B) the fact that we didn't penalize proteins with human orthologs (see Discussion subsection “No List is Canonical”). By adding two conditions to the weighted union to penalize proteins with orthologs in human and in mouse (with weights −40 and −20, respectively), some overlap between both lists can be observed: among our top 200 targets, 32 are also among the top 200 as ranked by Kumar *et al*. One unique aspect of our list is that it includes four tRNA synthetases among the top 39 proteins. These enzymes have been proposed as drug targets in *Brugia*, and are also considered good drug target candidates in other parasites such as trypanosomes [52], since they must be essential but often have major structural differences with respect to the human orthologs.

**Table 10 pntd-0000804-t010:** Rankings of possible *Brugia malayi* drug targets.

Ranking	Gene name	Gene product	Weight
1	Bm1_35945	Protein kinase domain containing protein	190
*2*	*Bm1_25205*	*leucyl-tRNA synthetase, putative*	*188*
2	Bm1_46445	vacuolar proton pump, putative	188
2	Bm1_31340	vacuolar proton pump, putative	188
5	Bm1_38680	tubulin alpha chain - mouse, putative	178
5	Bm1_20715	tubulin alpha-2 chain, putative	178
5	Bm1_25035	Tubulin alpha-2 chain, putative	178
5	Bm1_30720	KE2 family protein	178
5	Bm1_39900	Sex muscle abnormal protein 5, putative	178
*5*	*Bm1_32860*	*Valyl-tRNA synthetase, putative*	*178*
5	Bm1_14145	protein phosphatase PP2A regulatory subunit, putative	178
5	Bm1_44205	V-type ATPase 116 kDa subunit family protein	178
5	Bm1_48675	GTP-binding regulatory protein Gs alpha-S chain, putative	178
5	Bm1_28835	Transcription initiation factor IIA, gamma subunit, helical domain containing protein	178
5	Bm1_55400	Tubulin alpha-2 chain, putative	178
5	Bm1_46210	Protein kinase domain containing protein	178
5	Bm1_43680	T-complex protein 1, alpha subunit, putative	178
5	Bm1_30260	Tubulin alpha chain, putative	178
5	Bm1_44630	oxidoreductase, short chain dehydrogenase/reductase family protein	178
20	Bm1_10280	transketolase, putative	173
21	Bm1_20815	integrin-linked kinase, putative	170
21	Bm1_54155	Probable dimethyladenosine transferase, putative	170
23	Bm1_19675	Serine/threonine protein phosphatase F56C9.1 in chromosomeIII, putative	168
23	Bm1_50960	vacuolar ATP synthase catalytic subunit A, osteoclast isoform, putative	168
23	Bm1_52850	mannose-6-phosphate isomerase, class I family protein	168
*23*	*Bm1_12165*	*methionyl-tRNA synthetase, putative*	*168*
23	Bm1_48165	Adenosylhomocysteinase, putative	168
23	Bm1_32455	mannose-6-phosphate isomerase, class I family protein	168
23	Bm1_22825	Protein prenyltransferase alpha subunit repeat containing protein	168
23	Bm1_14125	proteasome subunit beta type 1, putative	168
23	Bm1_17330	succinate dehydrogenase [ubiquinone] flavoprotein subunit, mitochondrial, putative	168
23	Bm1_45960	ATP synthase beta chain, mitochondrial precursor, putative	168
23	Bm1_12875	Serine/threonine protein phosphatase PP1-beta catalytic subunit, putative	168
*23*	*Bm1_41830*	*Alanyl-tRNA synthetase, putative*	*168*
23	Bm1_38390	protein phosphatase 2A., putative	168
23	Bm1_24805	proteasome subunit beta type 3, putative	168
23	Bm1_41940	Glyceraldehyde 3-phosphate dehydrogenase, putative	168
23	Bm1_41510	FAD binding domain containing protein	168
23	Bm1_51640	Proteasome A-type and B-type family protein	168

Top targets according to the search criteria shown in Query 10 of Figure 2. Targets mentioned in the text are italicized.

**Table 11:** Rankings of possible *Schistosoma mansoni* drug targets.

Ranking	Gene name	Gene product	Weight
*1*	*Smp_015020*	*na+/k+ atpase alpha subunit, putative*	*120*
2	Smp_059790.2	transketolase, putative	119
2	Smp_059790.1	transketolase, putative	119
4	Smp_040790	cyclophilin B, putative	113
5	Smp_040970.1	vacuolar proton atpases, putative	112
*5*	*Smp_027920*	*tubulin alpha chain, putative*	*112*
*7*	*Smp_016780*	*tubulin alpha chain, putative*	*109*
*8*	*Smp_103140*	*alpha-tubulin, putative*	*106*
8	Smp_029390	ATP synthase subunit beta vacuolar, putative	106
10	Smp_106150	carbamoyl-phosphate synthase large chain, putative	103
*10*	*Smp_142050*	*erk1/2, putative*	*103*
10	Smp_071840	6-phosphogluconate dehydrogenase, putative	103
*10*	*Smp_030730*	*tubulin beta chain, putative*	*103*
14	Smp_059340.1	Guanine nucleotide-binding protein G(s) subunit alpha (Adenylate cyclase-stimulating G alpha protein), putative	102
*14*	*Smp_090120.2*	*alpha tubulin, putative*	*102*
*14*	*Smp_090120.1*	*alpha tubulin, putative*	*102*
14	Smp_059340.2	Guanine nucleotide-binding protein G(s) subunit alpha (Adenylate cyclase-stimulating G alpha protein), putative	102
14	Smp_043670.1	6-phosphofructokinase (Phosphofructokinase) (Phosphohexokinase), putative	102
*14*	*Smp_155270*	*hydroxymethylglutaryl-CoA synthase, putative*	*102*
*20*	*Smp_079960*	*tubulin beta chain, putative*	*100*
*20*	*Smp_035760*	*tubulin beta chain, putative*	*100*
*20*	*Smp_078040*	*tubulin beta chain, putative*	*100*
*20*	*Smp_079970*	*tubulin beta chain, putative*	*100*
24	Smp_165490	protein phsophatase-2a, putative	99
24	Smp_097590	valyl-tRNA synthetase, putative	99
24	Smp_096020.2	adenosylhomocysteinase, putative	99
24	Smp_096020.1	adenosylhomocysteinase, putative	99
24	Smp_028990.1	protein phosphatase-1, putative	99
24	Smp_096020.3	adenosylhomocysteinase, putative	99
24	Smp_028440.1	adenosylhomocysteinase, putative	99
24	Smp_028440.3	adenosylhomocysteinase, putative	99
24	Smp_034490	proteasome subunit beta type 6,9, putative	99
24	Smp_138590	hmg-CoA reductase, putative	99

Top targets according to the search criteria shown in Query 11 of Figure 2. Targets mentioned in the text are italicized.

The list of 57 recommended *Schistosoma* targets generated by Caffrey and colleagues (see Table S1 of [3], also available at http://tdrtargets.org/published/browse/247) includes 18 targets they considered to be of the highest priority because they are druggable, are expressed in relevant life-cycle stages, yield deleterious phenotypes, and are homologous to proteins with solved crystal structures including co-crystallized ligands. Of these 18 targets, eight rank within our 170 top *Schistosoma* targets. An obvious difference between the two lists is that ours includes nine tubulins among the top 23 proteins. The prominence of the tubulins is consistent with beta-tubulin's validation as a helminth drug target [53]. A number of ATPases also appear among our top targets. The top-ranked target in our list is the alpha (catalytic) subunit of a Na^+^/K^+^ ATPase (Smp_015020), which in mammals (and probably also in schistosomes) is the target of ouabain and other cardiac glycosides [54]. This target does not appear in the list of 57 targets published by Caffrey *et al.*; however, the beta subunit of this or a closely related Na^+^/K^+^ ATPase (Smp_124240) is ranked #52 in this study. Other attractive targets include a putative extracellular-signal-regulated kinase (ERK, Smp_142050), and a putative HMG-CoA reductase (Smp_138590), which is the target of cholesterol-lowering drugs like mevinolin [55].

## Discussion

### Stability of Ranked Lists

**Figure 3:**
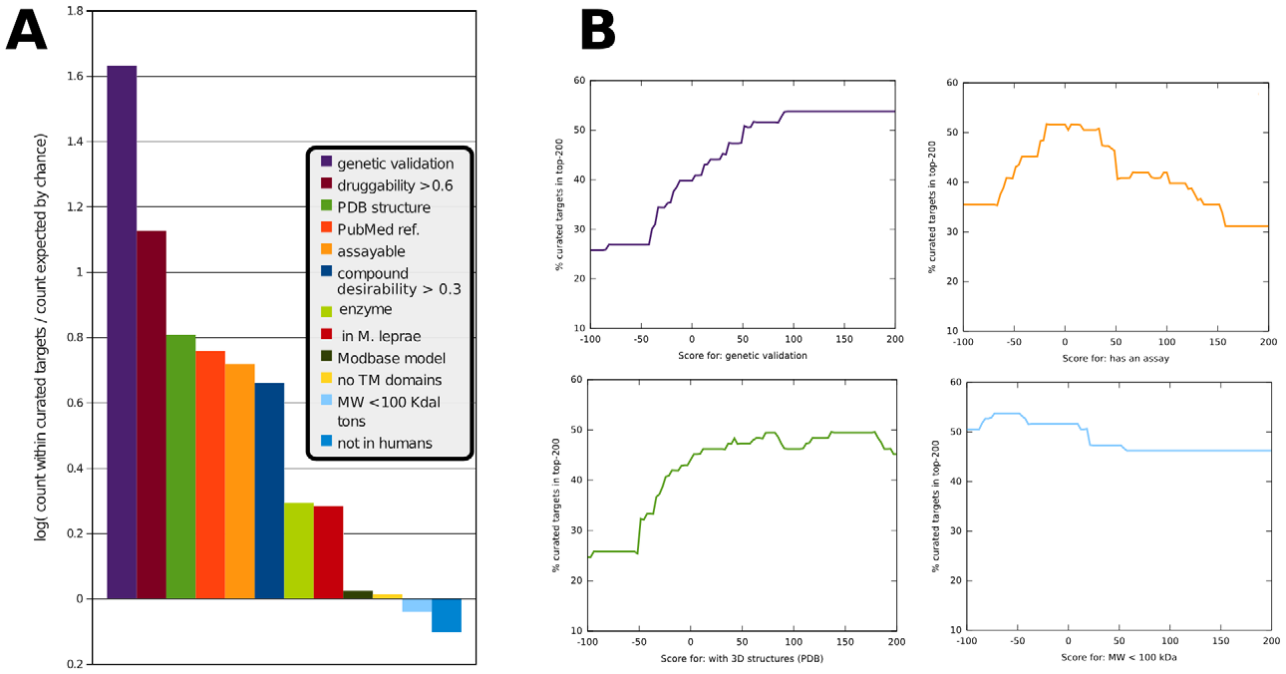
The sensitivity of target rankings to changes in weighting. Using the *M. tuberculosis* genome as an example, we determined the fraction of genes matching an attribute/query in a set of curated targets (validated chemically and/or genetically) and in the entire genome. (A) The results are shown for each attribute used in Query 5 of Figure 2. Values are log(Observed/Expected), where Expected is the fraction of genes in the genome that have the attribute and Observed is the fraction of curated targets that have the attribute. (B) We analyzed the stability of the final ranked list when the weight of a single attribute is changed. As an indication of stability, we plot the percentage of curated targets among the top 200 genes as the weight of each attribute is changed from minus-100 to plus-200.

A relevant question for any ranked list of targets using any strategy is: how different would this list be if the weight given to a certain attribute is changed? Using the *M. tuberculosis* queries whose results are in Table 5, we analyzed the robustness of the final ranked list, by selecting one attribute at a time and changing its weight from a very low (negative) score to a very high (positive) score. To assess the change observed in the ranked list we counted the number of curated targets (i.e., those with some level of validation) observed within the top 200 targets in the ranked list and used this value as our objective function (see panel B in Figure 3). Using this measure, we observed that a high score is obviously needed for those attributes that are enriched in validated targets (see panel A in Figure 3) in order to find well-known targets at the top of the list. This is also true for attributes that are not independent of these “good” attributes (e.g., availability of 3D structures). In contrast, changing the weight of attributes that are not expected to be enriched in validated drug targets (e.g., low molecular weight) does not affect the final result. In these cases, the final lists are all different, but they are consistent in having the highest ranks of the list being enriched in validated targets. In general, of course, targets' rankings within a list can be increasingly stabilized by including more and more relevant criteria in the prioritization.

### Old Targets Versus New Targets

In analyzing target candidates, we often wonder what sort of mix of well-studied and not-so-well-studied proteins might be most “desirable” at the top of a ranked list. On the one hand, having well-known targets or targets of known drugs at the top of our lists offers some assurance that our search strategies are reasonable (i.e., they serve as “positive controls” of the strategy). On the other hand, a method that only identifies well-established targets would not serve the important purpose of suggesting novel targets, so the presence of novel (even “hypothetical”) targets near the top of a list is also welcome. With the deliberate exception of Table 7, our lists reflect a desire to spotlight both previously validated and newly emerging targets.

In addition to trying to achieve a mix of new and established targets in prioritization lists, users need also to robustly consider which established targets they should classify as “successful.” Some targets enjoy long-held high esteem within the research community in the absence of any clinical validation, while other proteins, particularly for the organisms being studied here, are targets of clinically used drugs whose product profiles are unlikely to be acceptable in future drug development programs.

### False Negatives

Previous bioinformatic analyses of drug targets [4] have suggested that certain established targets never rank highly unless given artificial boosts in points for that specific purpose. Examples of these “false negatives” are also apparent in the lists presented here. For instance, cytochrome b is the known target of the antimalarial drug atovaquone [56], yet it ranks in the bottom 25% of targets represented by Table 4 because it has transmembrane domains (making recombinant expression difficult), is not easy to assay in isolation, lacks a known crystal structure, and so on. Likewise, certain targets of antihelminth drugs – such as the acetylcholine and GABA receptors, glutamate-gated chloride channel, and SLO-1 potassium channel [57], [58] – do not appear near the top of our helminth lists. There are several possible (non-exclusive) explanations for this. First, some drugs were found through phenotypic screens and their targets do not meet many of the criteria required in a target-based approach, and thus might not be expected to rank highly. Second, current target prioritization strategies are generally based on the assumption that drugs will cause a loss-of-function phenotype, but most existing helminth drugs lead to gain-of-function phenotypes [57]. Ranking proteins according to their potential as gain-of-function targets might be a fruitful direction of future work. Finally, it is conceivable that the total number of viable drug targets vastly exceeds the number that have been clinically validated, such that the position of many non-validated targets ahead of some validated ones is appropriate.

### False Positives

The failure of some validated targets to be highly ranked in our lists is not particularly surprising or troublesome, as discussed above. A more interesting issue is that of “false positives,” i.e., proteins that do rank highly but have not been validated as drug targets despite considerable effort. For instance, the *Leishmania* adenosine kinase ranks among the top 25 proteins in Tables 2 and 3, yet turns out to be nonessential in promastigotes [59]. Similarly, the *Plasmodium* enoyl-ACP reductase (FabI) ranks 2^nd^ in Table 4, yet is nonessential for blood-stage growth [60]. Among *M. tuberculosis* proteins, pantothenate kinase (PanK or CoaA) is in the top 100 of the Query 5 rankings (though not among the top 24 and thus not shown in Table 5), yet screens targeting this enzyme yielded no leads active against wild-type *M. tuberculosis* cells (C. E. Barry, personal communication). PanK activity *in vivo* appears to be so far in excess of what is required for growth that killing *M. tuberculosis* cells by inhibiting this enzyme is virtually impossible.

Although such examples can be seen as discouraging, we can use them to ask whether the incidence of false positives can be reduced through the use of additional datasets and search strategies. The nonessesentiality of the *Plasmodium* FabI during erythrocyte stages is perhaps suggested by the fact that expression of the enzyme is neither high nor tightly regulated during the erythrocyte life-cycle stages [61]. While TDRtargets.org does not currently offer a metric for the periodicity of gene expression in blood-stage *Plasmodium*, this could be added to future versions of the database.

### No List Is Canonical

The target rankings presented here are meant to be illustrative rather than definitive. The lists presented here were sent to experts on relevant neglected diseases for evaluation, and, predictably, we encountered numerous reasonable differences of opinion. For helminths, arguments were made both for and against penalizing proteins with orthologs in humans. The presence of human orthologs suggests an increased likelihood of toxicity in the host; on the other hand, several existing drug targets do have human orthologs. For *M. tuberculosis*, it was noted that existing drugs tend to target information-processing enzymes (DNA and RNA polymerase, DNA gyrase) rather than metabolic enzymes, so searches for new drugs might pay special attention to that area. Generally applicable suggestions included penalties for proteins that are part of macromolecular complexes, since they are hard to study in isolation, and for proteins of unknown function, since they are hard to study with biochemical or biophysical methods.

In addition to legitimate differences of opinion among researchers, the relative appeal of individual targets will continue to change as additional data are gathered. Fortunately, the infrastructure of TDRtargets.org is flexible enough to accommodate different individuals' interests (as seen especially in the lists focused on *T. brucei* glycolysis and *Plasmodium* apicoplasts) and the incorporation of new data (most prominent in the rankings for the helminths and for *M. tuberculosis* persistence). We therefore see TDRtargets.org as a tool that individual scientists may use to explore new research directions, rather than as a final arbiter of proteins' potential as drug targets.

As noted, target prioritization with TDRtargets.org or any other computational method is probably most useful as a prelude to (rather than a replacement of) laborious experimental follow-up work. Experimental characterization of promising targets often requires chemical inhibitors of target activity; therefore lists of target-specific inhibitors would be of great value to the research community. Though TDRtargets.org currently includes a preliminary dataset of such inhibitor-target associations, future editions of the database should offer major expansions and refinements of this dataset.

## Supporting Information

Alternative Language Abstract S1 Translation of the abstract into Spanish by Fernán Agüero.
(0.02 MB DOC)
