# Supplementary material for: Identification of Attractive Drug Targets in Neglected-Disease Pathogens Using an In Silico Approach
Source: PLoS Negl Trop Dis. 2010 Aug 24;4(8):e804. doi: 10.1371/journal.pntd.0000804 (PMC2927427; doi:10.1371/journal.pntd.0000804)
Supplement: Alternative Language Abstract S1 — Translation of the abstract into Spanish by Fernán Agüero. (0.02 MB DOC) [file pntd.0000804.s001.doc]

**Identification of attractive drug targets in neglected-disease pathogens**

**using an *in silico* approach**

**.**

Crowther GJ, *et al.*

**Resumen.**

**Antecedentes.** Se espera que el incremento en la secuenciación de genomas

de patógenos y la consiguiente disponibilidad de datos funcionales a

escala genómica guíen el trabajo experimental necesario para el

descubrimiento de drogas asociadas a blancos proteicos. Sin embargo, uno

de los mayores obstáculos en este emprendimiento ha sido la dificultad

de capturar e integrar información relevante en un formato fácilmente

accesible para identificar y priorizar potenciales blancos.

TDRtargets.org es un recurso de acceso libre que facilita el proceso de

priorización de blancos de drogas para los principales patógenos

causantes de enfermedades tropicales como las micobacterias

*Mycobacterium leprae* y *Mycobacterium tuberculosis*; los protozoarios

kinetoplástidos *Leishmania major*, *Trypanosoma brucei* y *Trypanosoma*

*cruzi*; los protozoarios apicomplejos *Plasmodium falciparum*, *Plasmodium*

*vivax* y *Toxoplasma gondii*; y los helmintos *Brugia malayi* y *Schistosoma*

*mansoni*.

**Metodología/Hallazgos principales.** En este trabajo presentamos

estrategias para priorizar proteínas de patógenos de acuerdo a la

evaluación de propiedades que son deseables en blancos para desarrollo

de drogas. Estos criterios están basados tanto en información derivada

de la secuencia (p.ej. peso molecular), datos funcionales sobre

expresión, esencialidad para el patógeno, fenotipos, vías metabólicas,

disponibilidad de ensayos bioquímicos y factibilidad de modulación

química (*druggability*). Esta aproximación pone de manifiesto el hecho de

que los datos para muchos de estos criterios no existen en patógenos

poco estudiados (p.ej. helmintos), y demostramos cómo esto puede ser

parcialmente solucionado a través del mapeo de información a partir de

genes ortólogos en organismos más estudiados. Además mostramos como

usuarios individuales pueden utilizar conjuntos de datos externos e

integrarlos con datos existentes en TDRtargets.org para generar listas

ranqueadas de potenciales blancos que se ajustan a criterios altamente

específicos.

**Conclusiones/Significancia**. Utilizando los conjuntos de datos y

herramientas disponibles en TDRtargets.org, hemos generado listas

ilustrativas de potenciales blancos para desarrollo de drogas en siete

patógenos que causan enfermedades tropicales. Aun cuando estas listas

son consistentes a grandes rasgos con los intereses de la

comunidad de investigadores sobre algunas proteínas específicas, y

sugieren a la vez nuevos blancos candidatos que ameritan ser estudiados,

las listas pueden ser fácilmente modificadas por los usuarios, ya sea

ajustando los pesos asignados a los criterios elegidos o cambiando los

criterios que fueron incluidos en cada caso.

**Translated by author** Fernán Agüero.
